# The complete mitochondrial genome of *Odorrana grahami* (Anura: Ranidae)

**DOI:** 10.1080/23802359.2021.1930217

**Published:** 2021-05-23

**Authors:** Yang Wen, Chunqing Li, Heng Xiao

**Affiliations:** a School of Life Sciences, Yunnan University, Kunming, P. R. China; b School of Ecology and Environmental Sciences, Yunnan University, Kunming, P. R. China

**Keywords:** *Odorrana grahami*, mitochondrial genome, full-length sequence

## Abstract

The mitochondrial genome of the Disckless-fingered Odorous Frog, *Odorrana grahami* (Anura: Ranidae), was sequenced using high-throughput sequencing technology. The genome length was 17864 bp, including 22 tRNA genes, 13 protein-coding genes, 2 rRNA genes and 1 control region (D-loop). The AT content of the mitochondrial genome was 55.9%. The composition of mitochondrial genome of *O. grahami* is similar to that of other species of the genus *Odorrana*. Phylogenetic analysis of the mitochondrial genomes of six congeners shows that *O. grahami* is sister to *O. margaretae*, but the analysis using 16S rRNA gene of additional congeners do not resolve their relationships.

*Odorrana grahami* (Boulenger. 1917) is part of an ancestral species group of the genus *Odorrana* (Anura: Ranidae; Chen et al. 2013), and it is diagnosed by most congeners by the lack of obvious adhesive pads at the end of the fingers (Boulenger. 1917). The species lives in small and medium-sized mountain streams at an altitude of 1720–3200 m, and it is mainly distributed in Sichuan, Yunnan, Guizhou, Shanxi, and Hunan Provinces of China (Fei et al. 2009, 2012; Chen et al. 2013). With the development of integrative taxonomic methods that utilize molecular genetic data, the taxonomy of Chinese amphibians has gone through major changes in the past decades (Wang et al. 2020), particularly of the genus *Odorrana* (Liu et al. 2021; Zhang et al. 2021), and the molecular genetic studies have revealed hidden evolutionary histories that were previously undetected (Qiao et al. 2018). However, the phylogenetic relationships among congeners remain unresolved in many cases (Liu et al. 2021), which is partly due to the lack of comparative genetic data and the limitation on the available genes. Here, we firstly reported the complete mitochondrial genome of *O. grahami*, which would better our understanding of the mitochondrial genome of the genus, help with the primers designs of mitochondrial genes of the genus *Odorrana*, and eventually facilitate the taxonomic and evolutionary researches of the group in the future.

We collected a sample of *O. grahami* (specimen SWFU 003918) from Daweishan National Nature Reserve, Pingbian Miao Autonomous County, Yunnan Province, China (N103°70′, E22°91′). The liver tissue was stored with 95% ethanol at −20 °C in the herbarium of Southwest Forestry University, Kunming, China (contact with Yang Wen, wengyang_wy@163.com). Genomic DNA of *O. grahami* was extracted using the DNAsecure Plant Kit (TIANGEN, Beijing, China). We used an Illumina HiSeq 2500 to perform paired-end sequencing of the sample DNA. After obtaining the sequencing data, the sequencing quality was first observed by FastQC tool (http://www.bioinformatics.babraham.ac.uk/projects/fastqc/) and NGSQC (Dai et al. 2010) software was used to quality control the sequencing data according to the observed sequencing quality. Then, using the SPAdes (version 3.9.0) software with the default parameter and no cut-off parameter, we splice all the scaffolds we could put together in clean data. This software mainly constructs contig based on DBG algorithm, interrupts read into Kmers, and splices multiple Kmers. Then, Price and Mitobim were used for extended merge stitching, and the number of iterations was selected to be 50. Finally, the mitochondrial genome was annotated by MITOS (http://mitos.bioinf.uni-leipzig.de/index.py) software (Bernt et al. 2013) and then submitted to GenBank (accession number MW551527).

The mitochondrial genome of *O. grahami* is a circular genome with a length of 17,864 bp. Including 22 tRNA genes (*trnH-GTG*, *trnE-TTC*, *trnS-GCT*, *trnR-TCG*, *trnG-TCC*, *trnK-TTT*, *trnD-GTC*, *trnS-TGA*, *trnY-GTA*, *trnC-GCA*, *trnN-GTT*, *trnA-TGC*, *trnW-TCA*, *trnM-CAT*, *trnQ-TTG*, *trnI-GAT*, *trnL-TAA*, *trnV-TAC*, *trnF-GAA*, *trnP-TGG*, *trnT-TGT*, and *trnL-TAG*), 2 rRNA genes (*rrnL* and *rrnS*), 13 protein-coding genes (PCGs) (*CYTB*, *ND6*, *ND5*, *ND4*, *ND4L*, *ND3*, *COX3*, *ATP6*, *ATP8*, *COX2*, *COX1*, *ND2*, and *ND1*) and 1 control region (D-loop) (Table 1). The composition of the mitochondrial genome of the *O. grahami* is similar to that of other species of the genus *Odorrana*, such as *Odorrana wuchuanensis* (Huang et al. 2016) and *Odorrana graminea* (Jin et al. 2020).

**Table 1.** The mitochondrial genome organization of *O. grahami*.

Gene	Strand	Start	End	Length (bp)	Spacer (+), Overlap (−)	Start codon	Stop codon
*trnH-GTG*	H	195	263	69			
*D-loop*	L	264	2483	2220			
*CYTB*	H	2484	3629	1146		ATG	TAG
*trnE-TTC*	L	3632	3700	69	+2		
*ND6*	L	3702	4202	501	+1	ATG	AGG
*ND5*	H	4259	6052	1794	+56	ATG	TAG
*trnS-GCT*	H	6085	6148	64	+32		
*ND4*	H	6167	7534	1368	+18	ATG	TAA
*ND4L*	H	7528	7812	285	−5	ATG	TAG
*trnR-TCG*	H	7813	7881	69			
*ND3*	H	7883	8221	339	+1	ATG	A
*trnG-TCC*	H	8222	8290	69			
*COX3*	H	8292	9074	783	+1	ATG	T
*ATP6*	H	9079	9750	672	+4	ATA	T
*ATP8*	H	9747	9914	168	−2	ATG	TAA
*trnK-TTT*	H	9915	9983	69			
*COX2*	H	9976	10,671	696	−6	ATG	AGA
*trnD-GTC*	H	10,672	10,740	69			
*trnS-TGA*	L	10,742	10,812	71	+1		
*COX1*	H	10,804	12,357	1554	−7	GTG	AGG
*trnY-GTA*	L	12,359	12,425	67	+1		
*trnC-GCA*	L	12,426	12,489	64			
*trnN-GTT*	L	12,518	12,590	73	+28		
*trnA-TGC*	L	12,591	12,660	70			
*trnW-TCA*	H	12,661	12,728	68			
*ND2*	H	12,730	13,761	1032	+1	ATT	TAG
*trnM-CAT*	H	13,762	13,830	69			
*trnQ-TTG*	L	13,830	13,900	71			
*trnI-GAT*	H	13,901	13,971	71			
*ND1*	H	13,972	14,917	946		ATG	T
*trnL-TAA*	H	14,919	14,992	74	+1		
*rrnL*	H	14,995	16,576	1582	+2		
*trnV-TAC*	H	16,577	16,645	69			
*rrnS*	H	16,646	17,580	935			
*trnF-GAA*	H	17,581	17,650	70			
*trnP-TGG*	L	17,652	17,720	69	+1		
*trnT-TGT*	H	17,721	17,789	69			
*trnL-TAG*	H	17,793	17,864	72	+3		

The AT content of the mitochondrial genome was 55.9%, and the base contents were: A 28.3%, C 15.5%, G 28.6%, T 27.6%, respectively. In addition to *ND6*, D-loop, and 8 tRNA genes (*trnE-TTC*, *trnS-TGA*, *trnY-GTA*, *trnC-GCA*, *trnN-GTT*, *trnA-TGC*, *trnQ-TTG*, and *trnP-TGG*), most of the genes in the mtDNA of *O. grahami* were distributed in the heavy (H) strand. Among the 13 PCGs in the mitochondrial genome, 10 genes (*CYTB*, *ND6*, *ND5*, *ND4*, *ND4L*, *ND3*, *COX3*

, *ATP8*, *COX2*, and *ND1*) have the start codon ATG, while the start codon of *ATP6*, *COX1*, and *ND2* genes are *ATA*, *GTG*, and *ATT*, respectively. In addition, 4 of the 13 PCGs (*CYTB*, *ND5*, *ND4L*, and *ND2*) used TAG as the stop codon, 2 genes (*ND6* and *COX1*) used AGG as the stop codon, 2 genes (*ND4* and *ATP8*) used TAA as the stop codon, and *COX2* used AGA as the stop codon. The *ND3* gene was terminated by an incomplete stop codon (single stop nucleotide A), and the other 3 genes (*COX3*, *ATP6*, and *ND1*) were terminated by single stop nucleotide T. Among the 13 PCGs, the shortest gene was *ATP8* (168 bp), and the longest gene was *ND5* (1794 bp). The length of 22 tRNA genes varied from 64 to 74 bp. The lengths of *rrnS*, *rrnl*, and D-loop were 935 bp, 1582 bp, and 2220 bp, respectively. The establishment of the complete mitochondrial genome of *O. grahami* will provide reliable genetic data for the further study of genetic evolution, phylogeographic structure, and molecular evolution of this species.

Mitochondrial genomes of seven species of Ranidae and mitochondrial 16S rRNA genes of eight species of *Odorrana* were downloaded from NCBI and used for phylogenetic analyses. *Rana omeimontis* and *Amolops wuyiensis* were used as the outgroups for mitochondrial genomes phylogenetic analysis, while *O. anlungensis* and *O. lungshengensis* were used as the outgroups for 16S rRNA phylogenetic analysis. Phylogenetic relationships were reconstructed using the maximum likelihood (ML) analysis based on the above two sets of genetic data, using RAxML (Stamatakis et al. 2008). Genetic data were partitioned by genes, and jModelTest 0.1.1 (Darriba et al. 2012) was used to calculate the optimal replacement model for each partition in the two sets of sequences respectively, which was GTR + G.

The resulting phylogenetic trees based on the mitochondrial genome suggest that *O. grahami* sister to *O. margaretae* (Figure 1(a)). Similar to previous studies (Liu et al. 2021), the results based on 16S rRNA gene sequences do not resolve the phylogenetic relationship of *O. grahami* with respect to *O. kuangwuensis*, *O. margaretae*, *O. andersonii*, *O. jingdongensis*, *O. wuchuanensis*, and *O. dulongensis* (Figure 1(b)).

**Figure 1.**
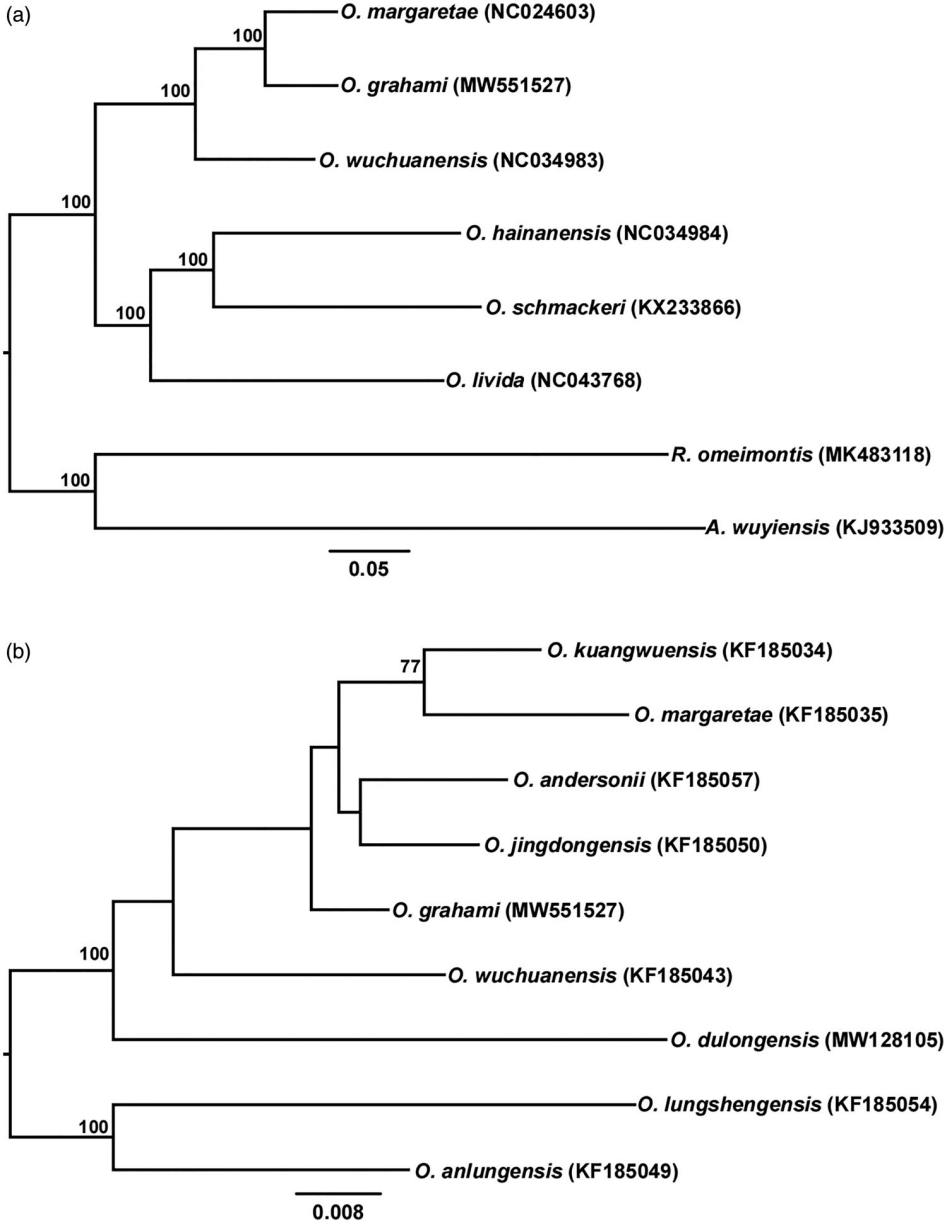
(a) Phylogenetic relationships of six *Odorrana* species based on available mitochondrial genomes using ML analysis. The values above branches represent bootstrap support values. The scale bar represents 0.05 nucleotide substitutions per site. *Rana omeimontis* (MK483118) and *Amolops wuyiensis* (KJ933509) were used as outgroups. (b) Phylogenetic relationships with an expended taxa sampling among closely related species of *O. grahami* inferred from 16S rRNA gene tree using ML analysis. The values above branches represent bootstrap support values. The scale bar represents 0.008 nucleotide substitutions per site. *Oodorrana lungshengensis* (KF185054) and *O. anlungensis* (KF185049) were used as outgroups.

## Data Availability

The genome sequence data that support the findings of this study are openly available in GenBank of NCBI at (https://www.ncbi.nlm.nih.gov/) under the accession no. MW551527.
